# Exceptional desiccation resistance in recalcitrant seeds of *Brosimum alicastrum* may confer ecological advantage

**DOI:** 10.1007/s00425-025-04910-y

**Published:** 2026-01-03

**Authors:** Louise Colville, Timothy R. Marks, Anaité López-Alquijay, Wolfgang Stuppy, Jayanthi Nadarajan, Hugh W. Pritchard, Alexandre Monro

**Affiliations:** 1 Royal Botanic Gardens, Kew, Wakehurst, Ardingly, West Sussex , RH17 6TN UK; 2 Instituto Nacional de Bosques, 7 Avenida 6-80 Zona 13, Guatemala City, Guatemala; 3 Kunming Institute of Botany, Chinese Academy of Sciences, Kunming, 650201 People’s Republic of China; 4 Department of Life Sciences, The Natural History Museum, Cromwell Road, London, SW7 5BD UK; 5 Royal Botanic Gardens, Kew, Richmond, Surrey, TW9 3AB UK; 6 Present Address: Ruhr-University Bochum Botanic Garden, Building BOTA 0/30, Universitätsstraße 150, 44801 Bochum, Germany; 7 Present Address: The New Zealand Institute for Plant and Food Research Limited, Private Bag 11600, Palmerston North, 4442 New Zealand

**Keywords:** *Brosimum alicastrum*, Cuticle, Desiccation, Germination, Recalcitrant, Seed

## Abstract

**Main conclusion:**

The recalcitrant seeds of *Brosimum alicastrum*, a widespread tropical tree, have an exceptional ability to resist desiccation, which we propose has contributed to the dominance of the species across a broad precipitation gradient in forests of Latin America.

**Abstract:**

Seed desiccation sensitivity is relatively common in tree species of tropical rain forests. For such species, pre-germination survival may be as important as seedling establishment in determining reproductive success, yet the adaptive traits important for survival are poorly understood. We studied seeds of *Brosimum alicastrum* Sw., a dominant tree species across a very broad precipitation gradient in Central America. This ecological success seems counterintuitive to the putative presence of seed desiccation sensitivity, that potentially severely limits propagule survival. We evaluated the anatomical, chemical and physiological traits for pre-germination survival in *Brosimum alicastrum*. Seeds were subjected to a series of desiccation experiments to determine the role of the seed coat and cuticular layers in controlling the rate of water loss. The structural properties were characterised using light and electron microscopy and complemented by biochemical and biophysical characterization of the seed cuticle. We confirm that *Brosimum alicastrum* seeds are highly desiccation sensitive but exhibit an exceptional resistance to desiccation. We show that the mechanisms for this trait of exceptional control of water loss are multifaceted and relate to the structural, biochemical and biophysical properties of the cuticle surrounding the embryo. When the cuticle is punctured, seed resistance to drying is lost and the seeds die rapidly. We propose that, combined with dispersal by winged fauna, this unique feature of seed desiccation resistance enables this species to colonise and occupy a broad range of edaphic and precipitation niches and so contribute to its prevalence in the forests of Latin America.

**Supplementary Information:**

The online version contains supplementary material available at 10.1007/s00425-025-04910-y.

## Introduction

Principal features of desiccation-sensitive (recalcitrant) seeds are being metabolically active throughout development and the requirement to retain a relatively high water content. Often dispersed at water contents c. 1 g H_2_O g^−1^ DW (i.e. moisture content of c. 50%), even small amounts of water loss may initiate viability loss (Pritchard 2004). The extent of desiccation sensitivity varies between species. In an analysis of 98 desiccation-sensitive species, the average lowest safe seed water content was reported to be 0.71 g H_2_O g^−1^ DW, whilst the absolute lowest safe seed water content is considered to be c. 0.25 g H_2_O g^−1^ DW (Tompsett and Kemp 1996; Hamilton et al. 2013). In contrast, desiccation tolerant (orthodox) seeds can survive desiccation to ≤ 0.1 g H_2_O g^−1^ DW (Walters 2015; Marques et al. 2018).

Desiccation sensitivity has consequences for recruitment success or failure (Joët et al. 2013) and is one of the main causes of mortality in situ, e.g. in *Quercus* species (Joët et al. 2016). Consequently, species with desiccation-sensitive seeds occur most frequently in aseasonal tropical moist forests where the risk of exposure to drought or freezing is low (Wyse and Dickie 2017). In environments with highly seasonal precipitation, recalcitrant seeds might be dispersed to coincide with the highest rainfall of the year, as found in a number of African tree species (Pritchard et al. 2004). Although non-deep dormancy is known in some recalcitrant seeds, such as the temperate tree *Aesculus hippocastanum* (Pritchard et al. 1999) and some palms (Jaganathan 2021), including *Mauritia flexuosa* (Veloso et al. 2016), such seeds are usually non-dormant and germinate relatively quickly post-shedding potentially reducing the risk from predators and competitors (Pritchard et al. 2004; Daws et al. 2005; Hill et al. 2012). However, a recent analysis has shown for trees that the thermal time for germination of recalcitrant seeds is slightly greater than that of orthodox seeds (*θ* = 149 vs 87 °Cd, on average for ≥ 25 species) (Pritchard et al. 2022). Contrary to orthodox seeds that might form a semi-persistent seed bank, non-dormant recalcitrant seeds are more likely to form a seedling bank under the forest canopy, which enables seedlings to respond swiftly to favourable conditions such as the formation of canopy gaps in shaded environments.

In addition to seedling establishment, pre-germination survival may be an important selection pressure for desiccation-sensitive seeds. Reducing the relative rate of water loss from recalcitrant seeds is facilitated by being larger (i.e. heavier; ≥ 1 g) than orthodox seeds (Hong and Ellis 1997; Dickie and Pritchard 2002; Tweddle et al. 2003; Daws et al. 2005, 2006). Being (near) spherical is another advantage, as the seed surface area to volume ratio is reduced; rarely are recalcitrant seeds flattened in shape (Tompsett and Kemp 1996; Hong and Ellis 1997). However, seed shape and size (or mass) do not account fully for pre-germination survival of desiccation-sensitive seeds. Other seed features are important determinants, including seed coat ratio (SCR) and desiccation rate (Hill et al. 2012).

Desiccation sensitive seeds of many woody floras, including Panama (Daws et al. 2005; 2006), Australia (Hamilton et al. 2013) and China (Lan et al. 2014), have been found to commit less resource to defence, as determined by a lower SCR (often ≤ 0.2), compared to orthodox seeds. Reducing the mass of the seed (sometimes fruit) covering layers has implications for the ease of germination but also the rate of water flow and thus survival time. In any particular environment, seed drying rates are highly dependent on mass and surface area to volume ratio. At the tissue level, there is uneven drying within embryos/embryonic axes (Pritchard et al. 1995; Ballesteros et al. 2014), potentially as a result of differences in cell wall thickness and stiffness (Subbiah et al. 2019a).

Thus, variation in seed tissue and cell morphology is likely to have a profound impact on the accumulation and interpretation of seed desiccation stress, including in the natural environment. Amongst *Cyclobalanopsis* species, retention of moisture in acorns is determined by the cupule scar vascular area of the pericarp (Xia et al. 2012). Similarly, multiple diaspore features of the recalcitrant-seeded species *Swartzia langsdorffii* slow desiccation so that seed viability is retained within the dispersed fruits for up to 7 months, until the rainy season begins (Vaz et al. 2016, 2018). In addition, seeds of *Garcinia cowa*, which are also dispersed during the dry season in seasonal rainforests, are protected by a hard tough coat (Liu et al. 2005). As a reduction in rainfall and dry season intensification threaten to increasingly expose recalcitrant seeds to the pernicious effects of drying (Hill et al. 2010; Pritchard et al. 2022), it is an imperative to understand the potential morphological adaptations in recalcitrant seeds that could underpin a survival strategy and thus continuing species success.

The focus of this study is the widespread tropical forest tree species, *Brosimum alicastrum* (Lander and Monro 2015; Gardner et al. 2021), a member of the Moraceae (fig family). *B. alicastrum* is a late-succession canopy emergent tree and a widespread and frequently dominant species on shallow substrates incorporating limestone, but also found on lateritic and volcanic soils (Peters and Pardo-Tejeda 1982) in evergreen, semi-evergreen, semi-deciduous and seasonally dry tropical forests in Mexico and Central America but with a range which extends to South America north of the Amazon river (Sosa et al. 1975; Rzedowski 1978; Kammesheidt 2000; Pennington and Sarukhán 2005; Mueller et al. 2010). The mature fruit of *B. alicastrum* consists of a single-seeded achene embedded in the edible fleshy tissue of the peduncle. The fruits are eaten and the seeds dispersed by bats (García-Morales et al. 2012), parrots (Monterrubio-Rico et al. 2009) and monkeys (Cristóbal-Azkarate and Arroyo-Rodríguez 2007). Fresh seeds have a high moisture content (57% w/w) and have been proposed as desiccation sensitive by several authors (Pence 1995; Gillespie et al. 2004; Román et al. 2012). However, relations between seed structural features and moisture flow characteristics of *B. alicastrum* seeds have not been thoroughly investigated hitherto.

The aim of this study was to evaluate traits for pre-germination survival in *B. alicastrum* and identify their anatomical and physiological bases. The findings provide an answer to a conundrum in plant and seed ecology, in which a species with recalcitrant seeds has become prevalent in the forests of Latin America with a broad range of edaphic and precipitation niches.

## Materials and methods

### Seed source/sampling

A batch of ca. 5000 seeds was obtained from > 20 individuals of a single population of *Brosimum alicastrum* from the Ixlú community (16° 58′ 19″ N 89° 41′ 9″ W), Flores, Petén, Guatemala in September 2012. Seeds were collected from the forest floor, as is typical for seed collection from tall, tropical trees (Gillespie et al. 2004; Vozzo 2002). For most seeds on the forest floor, the fleshy pulp had already been removed by bats, birds, monkeys or other large animals in the canopy. After collection, the seeds were washed in water, dried in ambient conditions for 6 h before being packed in paper bags and shipped to the Royal Botanic Gardens, Kew, Wakehurst where this work took place. Shipping took 7 days. Twelve voucher seedlings were generated from this batch and are accessioned in the Jodrell Laboratory Greenhouses at Royal Botanic Gardens, Kew (accession number: 672205).

An additional batch of ca. 40 *B. alicastrum* seeds of Guatemalan provenance was obtained from trees in cultivation at the Montgomery Botanical Centre, Florida, USA in March 2015. This was used to assess the role of cuticular wax in controlling the rate of desiccation. All seeds were stored in a controlled environment room at 15 °C and 75% RH prior to use.

### Determination of the seed coat ratio

Ecological correlates used to predict desiccation sensitivity include a combination of the seed coat ratio (SCR) and seed mass (Daws et al. 2006). Ten seeds were sampled to determine SCR using the methodology of Daws et al. (2006).

### Identification of optimal germination temperature

To assess the optimal temperature for germination, three replicates of 10–15 seeds were sown on 1% (w/v) water agar in a plastic box and incubated under eight temperature regimes from 5 to 40 °C with an 8/16 h photoperiod (light/dark). The selected temperature range encompasses the mean annual minimum and maximum temperatures in Guatemala ± 10 °C. Cumulative germination was assessed every day for up to 27 days. Cumulative germination data were used to produce values for maximum germination at each temperature, and the time for 50% of the seeds to germinate (*t*_50_) was determined by fitting a logistic regression model.

### Determination of optimal temperature for seed storage

To determine the optimal temperature for storage, 3 replicates of 20 fresh seeds were sealed in separate zip-lock polythene bags and placed in incubators at 5, 10, 15, 20 and 25 °C under an 8/16 h photoperiod, for 4 and 10 weeks. The bags were opened every 7 days to allow an exchange of air. After both periods of storage, ungerminated seeds were removed and sown as previously described at 30 °C. The occurrence of germination both during storage and post-storage was recorded.

### Seed desiccation experiments

The relationship between seed moisture content (MC) and germination success was assessed by equilibration of the seeds to three RH regimes at 15 °C: ca. 5% RH (over regenerated silica gel), 15% RH (dry room) and 75% RH (hydration room). Shrinkage and associated wrinkling of the epidermis was observed in some seeds stored at 5 and 15% RH, which suggested differential water loss between individual seeds. Initially shrunken seeds were therefore excluded from further measurements. Seeds were periodically removed from each treatment and seed MC and germination were measured. Germination was assessed using three replicates of 15 seeds at 30 °C as described above, with the exception that decayed and mouldy seeds were removed from the test and germination was reported as a percentage of germinated and intact non-germinated seeds remaining at the end of the test, rather than the number of seeds sown. Seed MC of five replicates of single seeds was determined gravimetrically following oven-drying for 17 h at 103 °C and using the differential between fresh and dry weights to calculate weight loss attributed to water content.

### Assessment of water loss and identification of the mechanisms responsible for its control

*Brosimum alicastrum* seeds were differentially scarified to assess the effect of mechanical damage upon overall moisture content. Four treatments were applied to replicates of five seeds: (1) seeds were left intact; (2) the seed coat was carefully removed to address control of water flow by the seed coat; (3) two 1 cm long longitudinal cuts (> 2 mm) were made into each cotyledon, four cuts in total, to breach the cuticle and address the control of water flow through the cuticle; and (4) the surfaces of both cotyledons were abraded with sandpaper to expose an area of ca. 1 cm^2^ on each as an alternative mechanism for breaching the cuticle. All seeds were then exposed to 15 °C and 15% RH and weighed three times a week over a period of 60 days. At the end of the experiment, the dry weight of the seeds was measured after oven-drying (as described above) to enable the MC of the seeds throughout the experiments to be calculated.

A separate experiment was performed to compare the rate of desiccation of intact and surface abraded seeds with seeds from which the cuticular wax had been removed. Surface abrasion was performed as described above with the exception that only the surface of one cotyledon was abraded. Cuticular wax was removed by three sequential washes of 30 s in chloroform. Seeds were stored at 15 °C and 15% RH and three seeds for each treatment were individually weighed daily over a period of 24 days. After this time, the seeds were oven-dried to determine their MC. Eight abraded seeds were removed after 0, 2, 4, 8 and 24 days and sown for germination testing as described above. Germination testing of intact and de-waxed seeds was performed only after 24 days of drying.

### Seed anatomy

Fresh seeds were sectioned at a thickness of 18 µm at −15 °C using a cryo-microtome (Leica CM3050S, Leica Microsystems, Wetzlar, Germany). Sections were placed on microscopic slides and subsequently freeze-dried inside the cold chamber of the cryo-microtome. For differentiating cuticles, sections were stained with 5% (w/v) Sudan IV in 70% ethanol and mounted in glycerine. Photomicrographs were taken using a Leitz Diaplan photomicroscope with a Leitz digital camera.

For scanning electron microscopy (SEM), the seeds were dissected into smaller samples in 70% ethanol, and then dehydrated through absolute ethanol and critical-point-dried using a Balzers CPD 020 (Kew). They were dissected and mounted onto specimen stubs using double-sided conductive adhesive tabs (Carbon Tabs, Agar Scientific), coated with platinum using a Quorum Q150T Sputter Coater, and examined using a Hitachi S-4700-II cold field emission SEM at a voltage of 2.0 kV and secondary electron detector at the Royal Botanic Gardens, Kew.

### Biochemical and biophysical characterisation of cuticular layer

#### Surface wax and soluble lipid extraction and derivatisation

Five replicates of three seeds were incubated in 20 mL of chloroform: methanol (1:1, v/v) for 7 days. The chloroform:methanol solvent was changed daily, and the extracts were pooled and dried under nitrogen. The dried extracts were derivatised with *N*,*O*-bis(trimethylsilyl)trifluoroacetamide (BSTFA; Fluka Analytical) in pyridine (1:1, v/v) at 70 °C for 60 min. The derivatised extracts were then dried under nitrogen and dissolved in 1 mL chloroform prior to GC–MS analysis (modified from Molina et al. 2006).

#### Cutin polymer isolation

Following the removal of cuticular waxes, the seeds were incubated in citric acid (0.079 M)–sodium phosphate (NaH_2_PO_4_, 0.04M) buffer (pH 3) containing 1% (w/v) pectinase (Sigma-Aldrich) for 2 weeks at room temperature, with a change of buffer after 1 week. The cutin polymer was collected and washed for 5 days with 100 mM borate buffer (pH 9), which was renewed once to remove lipophilic compounds. The cutin was then rinsed three times with distilled water and freeze-dried (Franke et al. 2005).

#### Cutin depolymerisation

Based on a method described by Franke et al. (2005), dried cuticles were incubated with 5 mL 1 N methanol/HCl at 80 °C for 2 h. 2 mL of saturated sodium chloride solution was added and the hydrophobic monomers were extracted three times with 2 mL hexane. The combined hexane extracts were pooled and dried at 30 °C under nitrogen and derivatised as described above with the addition of 10 μL of 10 mg mL^−1^ heptadecanoic acid (Fluka Analytical) in pyridine as an internal standard.

#### GC–MS analysis of cutin and cuticular wax components

1 μL of derivatised extract was injected onto the GC (Thermo Finnigan Trace GC Ultra, Thermo Fisher Scientific, Waltham, USA), and components were separated using a fused silica SPB-50 capillary column (30 m length, 0.25 mm internal diameter, 0.25 μm film thickness; Supelco) with a temperature program (5 min at 70 °C, 5 °C min^−1^ ramp to 310 °C, 5 min hold at 310 °C for cutin analysis or a 15 min hold at 310 °C for wax analysis, 70 °C min^−1^ ramp to 70 °C, 6 min hold at 70 °C) and helium carrier gas at a constant flow rate of 1 mL min^−1^. The injector temperature was 230 °C and a split injection (10:1) was performed. The compounds were detected using MS (Thermo Finnigan Trace DSQ; ionisation energy 70 eV, scan frequency range *m*/*z* 50–600 per 0.2 s; ion source temperature 200 °C) and identified through comparison with the National Institute of Standards and Technology (NIST, Gaithersburg, MD, USA) mass spectral database.

#### Differential scanning calorimetry of cutin and cuticular wax

Differential scanning calorimetry (DSC) was used to characterise the biophysical properties of endothermal and exothermal phase transitions of cutin and cuticular waxes with a DSC 1 (Mettler-Toledo, Switzerland) and a TC125-MT Intracooler (Huber, Germany) controlled by STARe software (version 12, Mettler-Toledo, Switzerland). The DSC was calibrated with indium and zinc as standards.

The cuticular wax was extracted as described above, but individual seeds were incubated in 10 mL of chloroform:methanol solution for 2 days. The cutin was isolated as described above with the following modifications: the incubation time in the pectinase solution was reduced to 6 days with the solution changed once after the first day, and the cutin was washed in borate buffer overnight. Tissues weighing between 1 and 5 mg (cutin) and 16–44 mg (cuticular wax) were placed in pre-weighed aluminium pans and non-hermetically sealed using a crucible sealing press (Mettler-Toledo, Switzerland) and the fresh weight values recorded. Samples were cooled from 25 to -80 °C and then rewarmed to 80 °C at a cooling/warming rate of ± 10 °C min^−1^. To establish a baseline, the programme was carried out on an empty pan. The heat capacity (Δ*Cp*) of glass transition and enthalpies (ΔH) of associated lipid melting endotherms were calculated using the STARe software. Five replicates were used for all thermal analyses.

### Statistical analysis

Germination and moisture content data were arcsine transformed to normalise values and were analysed using either one- or two-way ANOVA (Genstat software, Version 12 VSN International, 2011). The results of which are shown as non-transformed values in the figures. These values were compared using Duncan’s multiple range test, significant at *P* ≤ 0.05. Logistic regression curves were fitted to cumulative germination data to allow the calculation of the 50th percentile of germination, and linear and exponential regression curves were fitted to seed drying data following scarification using the statistical function of Origin, Version 9 (OriginLab Corporation, USA).

## Results

### Traits identified for pre-germination survival: desiccation sensitivity based on the seed coat ratio

The mean fresh seed mass was 3.07 g ± 0.44 (SD) per *B. alicastrum* seed with a range of 2.24–4.03 g across 20 seeds sampled. Based on the paired dry weights of embryos and their seed coats, the probabilistic seed coat ratio–seed mass (SCR-SM) model (Daws et al. 2006) gave a value for *P* of 0.97 ± 0.008. Predicted probability (*P*) values of > 0.5 indicate that seeds are more likely to be desiccation sensitive than tolerant, hence a value of 0.97 strongly suggests that *B. alicastrum* seeds can be classified as desiccation sensitive (Supplementary Table 1).

### Traits identified for pre-germination survival: optimal germination temperature

**Fig. 1:**
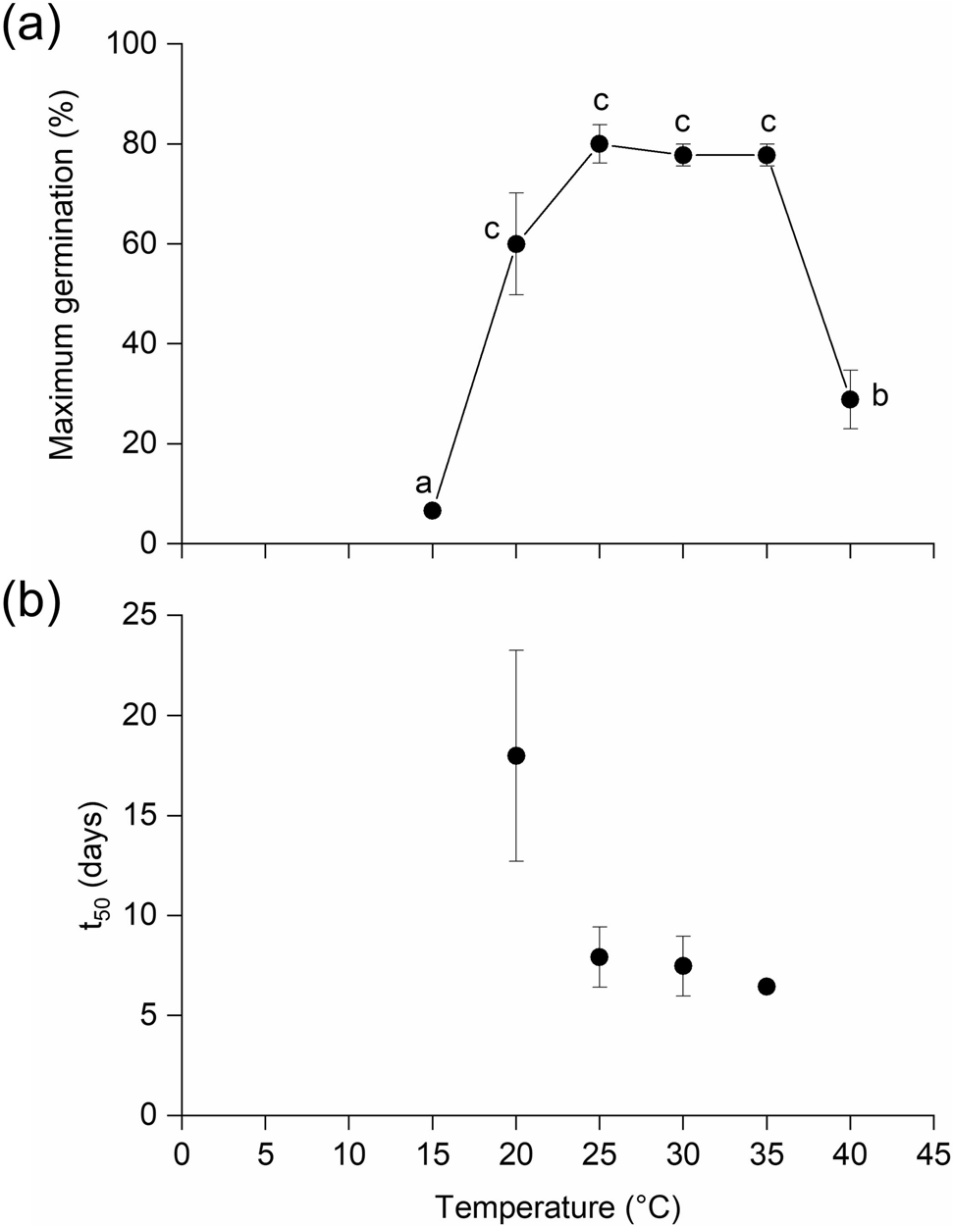
Comparative analysis of germination at 15–40 °C; maximum germination achieved after 25 days (**a**); plot of time to attain the 50th percentile of final germination (*t*_50_) (**b**). Error bars are standard errors of treatment means. Letters indicate significant differences (*P* < 0.05) between treatments

Germination testing at temperatures from 5 to 40 °C was performed to determine the optimum temperature for *B. alicastrum* seed germination. Cumulative germination reached a maximum of ca. 80% after 25 days at 25, 30 and 35 °C (Fig. 1a, Supplementary Fig. 1). This was significantly higher (*P* < 0.001) than at 15, 20 and 40 °C. No germination occurred at either 5 or 10 °C. The time taken for 50% of seeds to germinate (t_50_) was greatest at 20 °C at ca. 22 days compared to ca. 7 days at 25–35 °C (Fig. 1b).

### Traits identified for pre-germination survival: optimal storage temperature

**Fig. 2:**
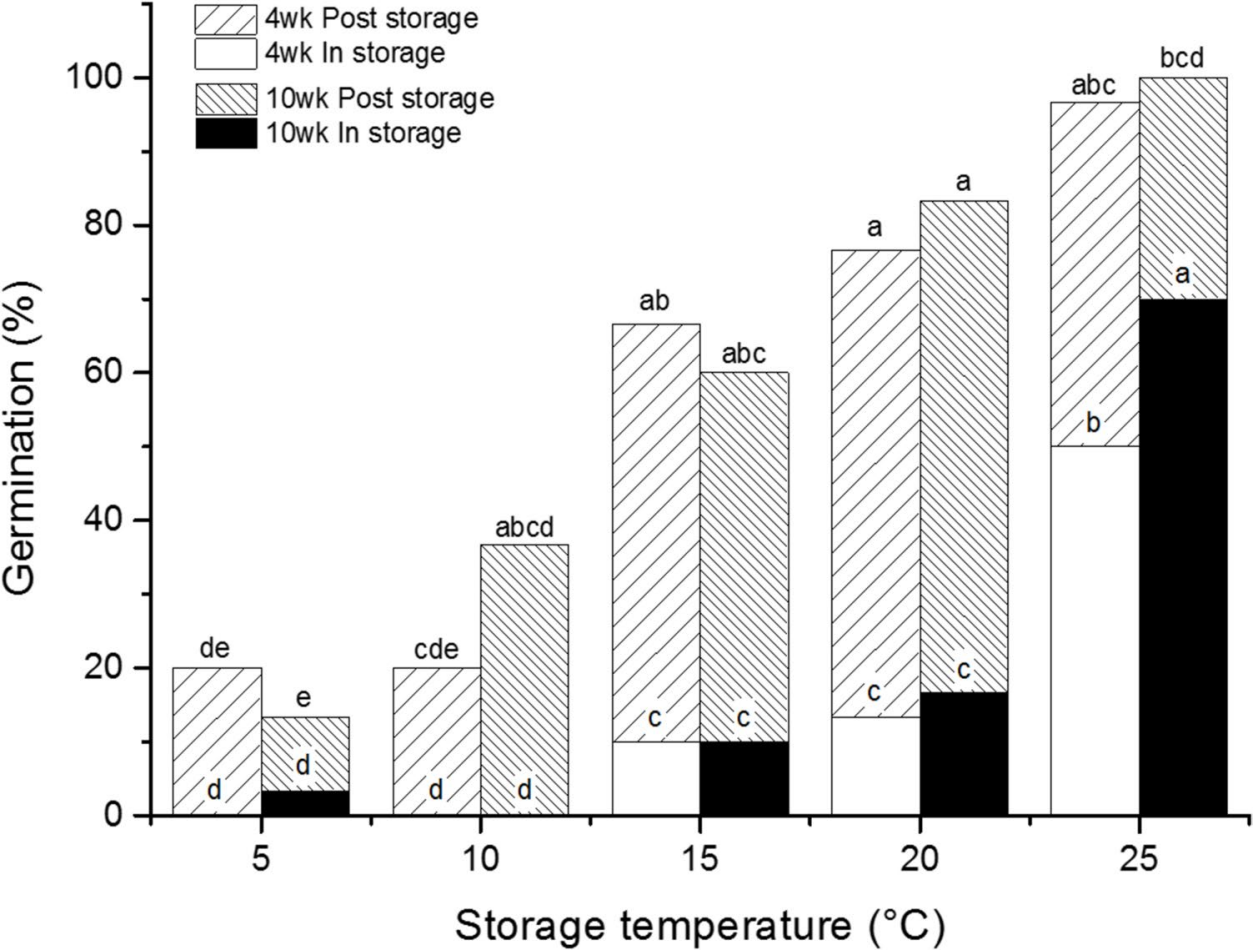
Germination observed during storage for 4 and 10 weeks at temperatures of 5–25 °C and post-storage on 1% agar at 30 °C. Different letters indicate significant differences (*P* < 0.05) in germination during storage or post-storage between storage temperatures and storage time

Seeds stored at five temperatures for 4 and 10 weeks showed a similar proportional and significant (*P* < 0.001) relationship to increasing germination during storage with increasing temperature. This amounted to the loss of 50–70% of seeds at 25 °C, but only 10% at 15 °C (Fig. 2). However, the effect of length of storage time upon the level of germination during storage was only significant at 25 °C. The percentage of seeds germinating post-storage was very similar for seeds stored at 15–25 °C, but seeds stored at 5 and 10 °C showed much lower post-storage germination and there was considerable fungal contamination on the agar medium. Fungal growth was almost absent at the three higher temperatures.

### Experimental determination of seed desiccation sensitivity

**Fig. 3:**
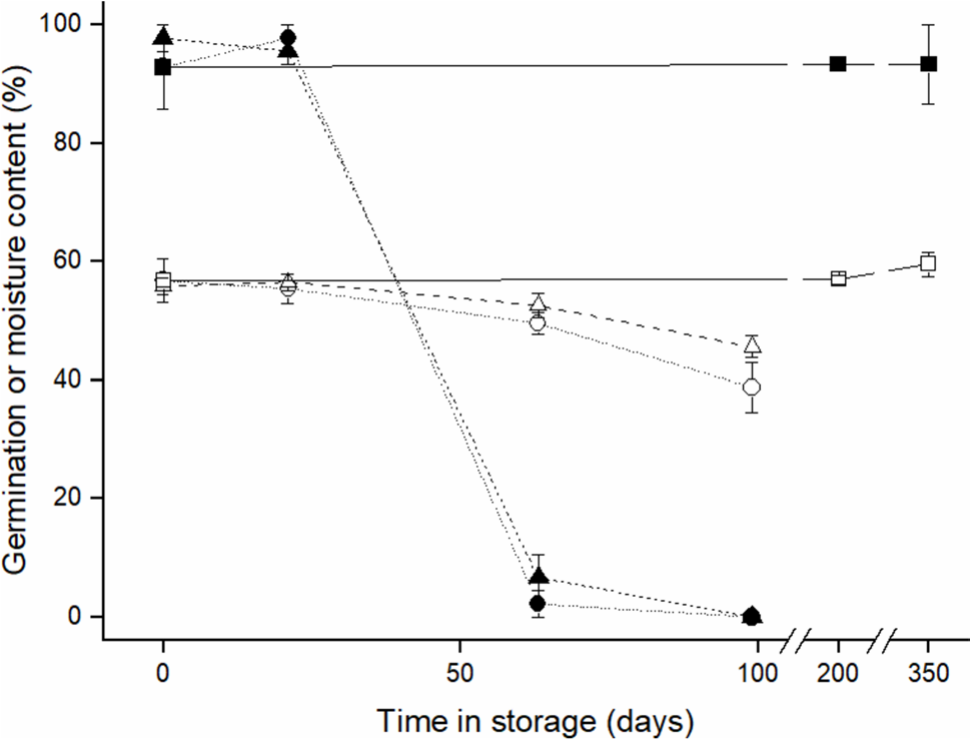
Germination at 30 °C (solid symbols) and moisture content (open symbols) following storage at 15 °C and either 5 (circles), 15 (triangles) or 75% (squares) relative humidity. Error bars are standard errors of treatment means

*Brosimum alicastrum* seeds were stored at three relative humidities, 5, 15, and 75% RH, for up to 350 days to determine the effects of desiccation on the seed MC and germination. Intact seeds stored at 75% RH and 15 °C did not show a significant change in either MC or germination success at 30 °C over the 350 day storage period (Fig. 3). Seeds maintained at two, low relative humidities (5 and 15%) at 15 °C showed minimal drying and no significant change in germination or MC over 25 days (Fig. 3) but subsequent measurements showed a steady decrease in MC after 63 days (n.s.) and 99 days (*P* < 0.05) with minimal or no germination, respectively.

### Assessment of the factors controlling desiccation

**Fig. 4:**
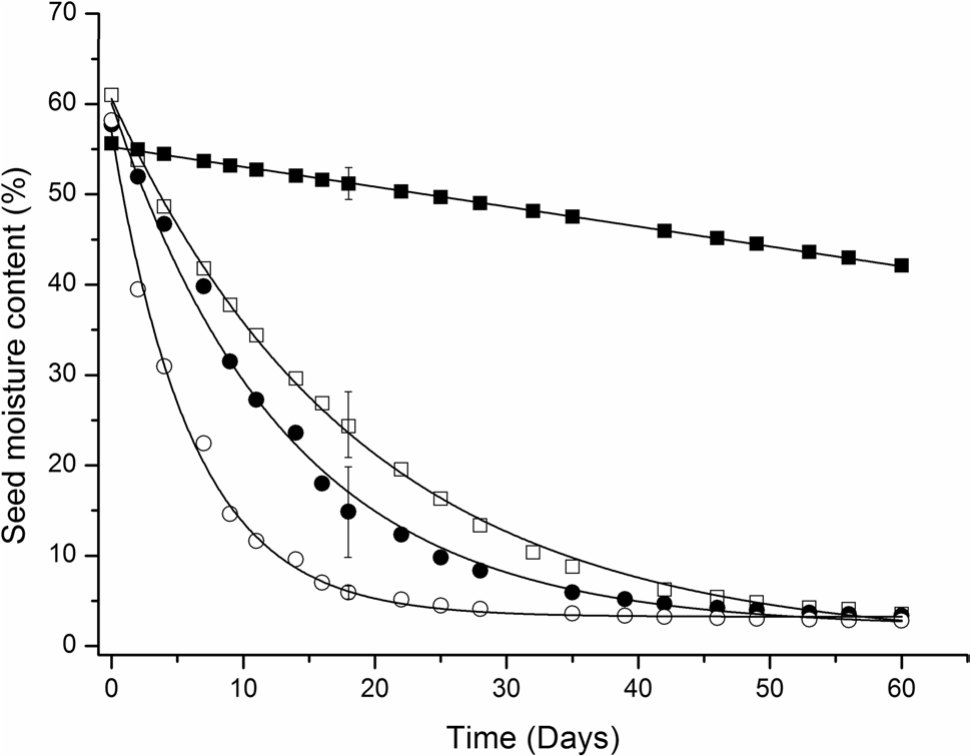
Linear and exponential drying response curves of intact and differentially scarified seeds; intact seed (closed squares), seed coat removed (open squares), longitudinally cut (closed circles), surface abraded (open circles). For clarity, only single illustrative standard error bars of treatment means are shown

**Fig. 5:**
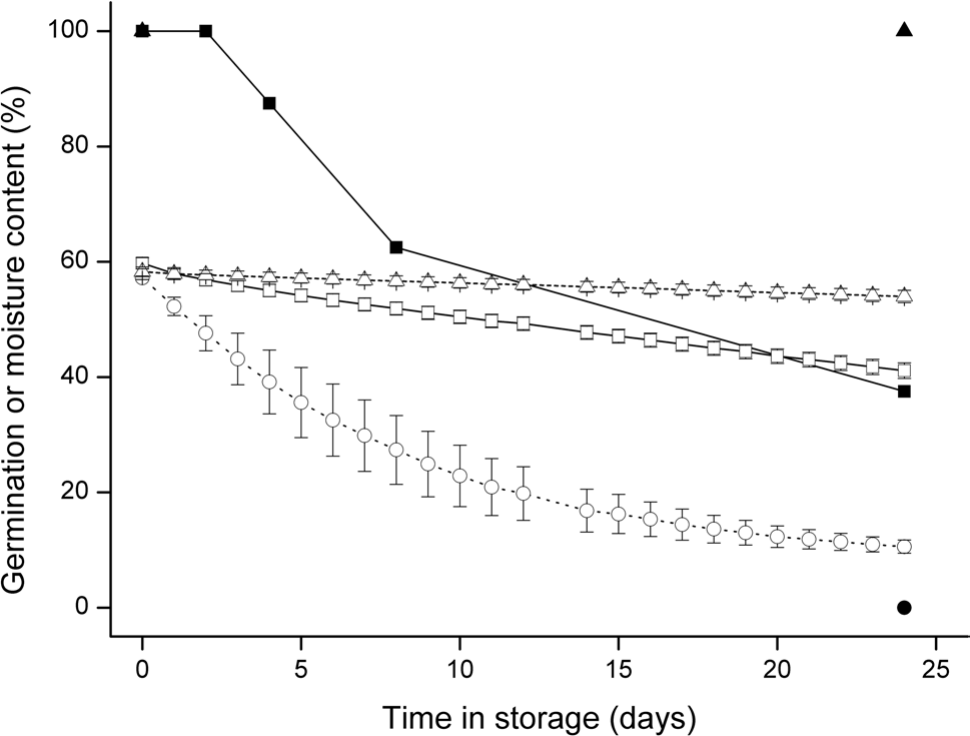
Germination (solid) and moisture content (open) following storage of intact (triangles), surface abraded (squares) and de-waxed (circles) seeds at 15 °C and 15% RH. Error bars are standard errors of treatment means

The roles of the seed coat and the embryo epidermis and cuticle in controlling desiccation were assessed through comparing desiccation rate in intact seeds with seeds following seed coat removal, surface abrasion of the epidermis, or removal of the cuticular wax. The rate of desiccation at 15% RH was greatly accelerated by the removal of the seed coat (where minimal damage resulted in exudation of latex from the cotyledons) or through cutting or abrading the epidermis. Over a 60-day period, the MC of intact seeds declined from 55.6 ± 1.00 to 40.6 ± 4.67%, whilst the MC of scarified seeds was reduced to 3.2 ± 0.48% (*P* < 0.001) (Fig. 4). In a complementary experiment, the rate of desiccation was higher in surface abraded seeds compared to intact seeds, and this resulted in a decline in germination within 4 days of storage at 15% RH and 15 °C. Removal of the cuticular wax with chloroform greatly accelerated the rate of desiccation with the MC falling from 57.2 ± 0.31% to 10.6 ± 1.14% within 24 days compared to 54.0 ± 1.06% for intact seeds and 41.1 ± 1.34% for abraded seeds. Total germination after 24 days was 100, 38 and 0% for intact, surface abraded and de-waxed seeds, respectively (Fig. 5).

### Seed and cuticle anatomy

**Fig. 6:**
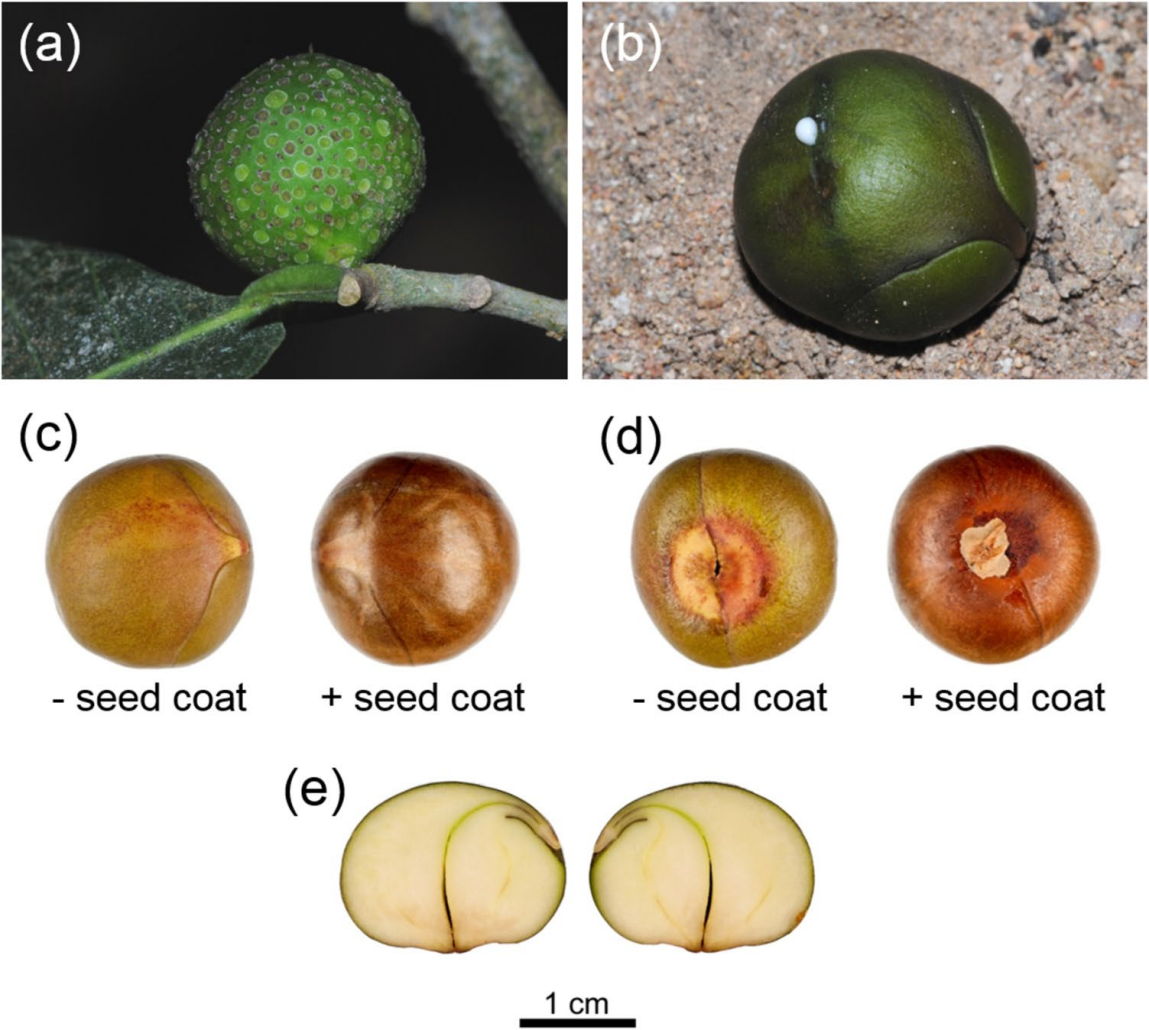
*Brosimum alicastrum* morphology. Fresh mature fruit/monocarpic infructescence, which consists of a single-seeded achene or nut embedded in the edible fleshy tissue of the peduncle. The surface is covered in the remains of the bracteoles associated with aborted male flowers (**a**). Seed with fruit and seed coat removed showing latex exudation following minor damage to the cuticle (**b**). Comparison of seed with and without the seed coat (**c**, **d**). Longitudinal section through the seed showing the very thin seed coat and the externally placed embryonic axis and the two enlarged cotyledons (**e**)

**Fig. 7:**
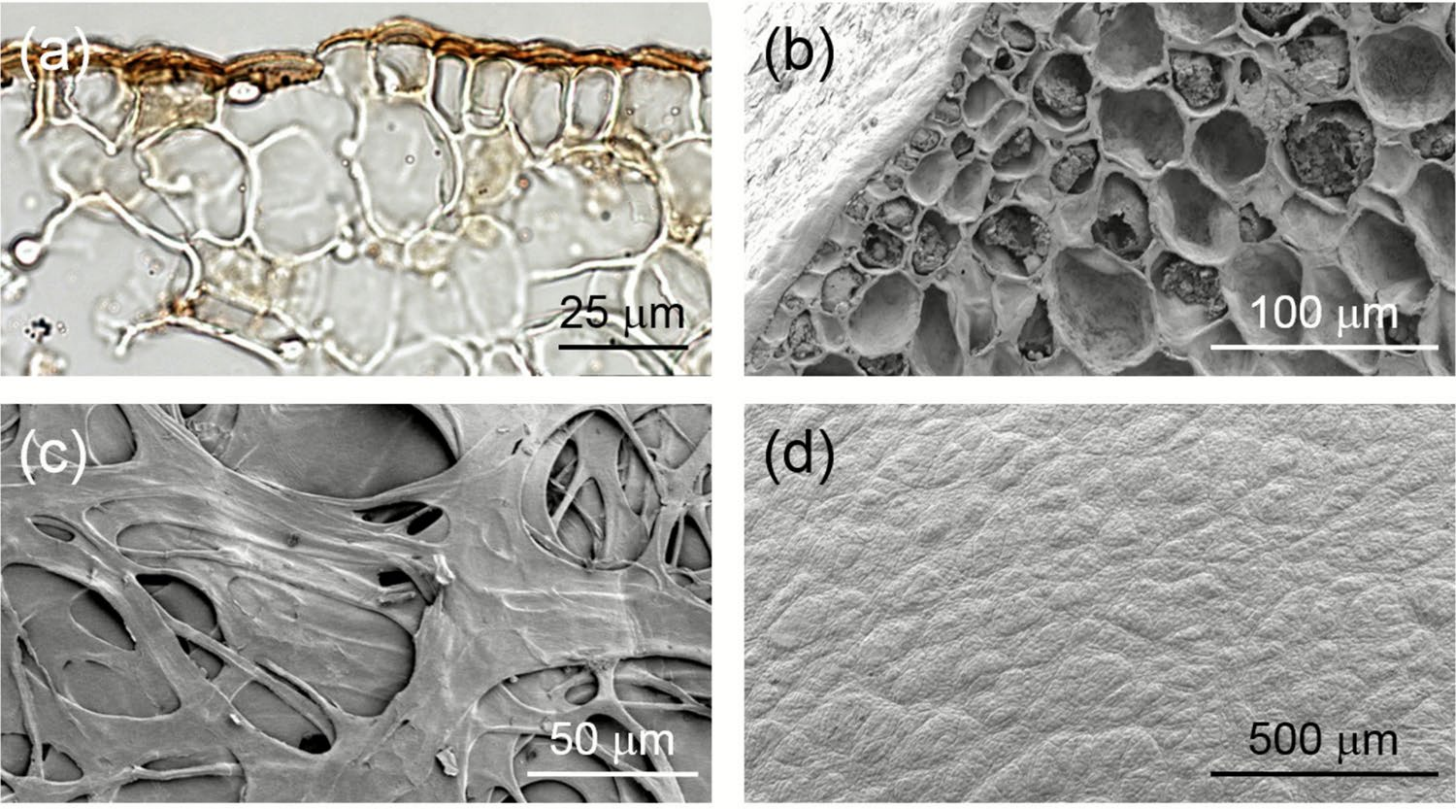
Micromorphological details of the embryo epidermis and seed coat of *Brosimum alicastrum*: **a** Cross-section through cotyledon; the epidermis of the cotyledon is covered in a prominent cuticle (stained red with Sudan IV); **b**–**d** scanning electron micrographs showing: **b** a cross-section through the cotyledon with the smaller celled epidermis on the left; **c** the inner surface of the seed coat displaying a lattice-like structure with many large perforations (originally probably intercellular spaces) in the different layers; **d** surface of the embryo with the epidermis completely devoid of stomatal openings

The mature fruit consists of a single-seeded achene embedded in the edible fleshy tissue of the peduncle, the latter resembling the fleshy pericarp of a berry or drupe. The surface of the spherical peduncle is covered in the remains of the bracteoles that used to protect the male flowers. The actual pericarp forms a very thin, parchment-like layer contiguous with, but not attached to, the seed. The endosperm-less seed contains a bent storage embryo with enlarged cotyledons joined by a short embryonic axis (Fig. 6). The actual seed coat is represented by a very thin, brown skin that consists of several layers of collapsed, brown, thin-walled cells (Fig. 7). There are no mechanical elements (i.e. thick-walled cells) in the seed coat and significant cuticles are missing. The only structural feature which could explain the seed’s extreme resistance to desiccation is the prominent cuticle (Fig. 7a) that covers the embryo’s epidermis and ranges in thickness between 2.5 and 6 μm, similar to the values between 0.1 and 10 μm reported for plant cuticles (Riederer and Schreiber 2001).

### Biochemical composition of cutin and cuticular waxes

The chemical composition of the isolated cutin was determined using GC–MS. The major components were straight-chain fatty acids with chain lengths ranging from 16 to 24 carbons. Palmitic, oleic and linoleic acids were the dominant fatty acids. Six 2-hydroxy fatty acids were detected, of which five were very long-chain (> C_22_), and only one was mid-chain (C_16_). No ω-hydroxy fatty acids were detected. Other compounds detected were three triterpenoids (α-sitosterol, α-amyrin and lupeol acetate), a fatty alcohol (octacosanol, C_28_) and α-tocopherol (Table 1, Supplementary Fig. 2). Solvent extraction of the cuticular waxes followed by GC–MS identified the wax components as comprised mainly triterpenoid compounds, particularly phytosterols including sitosterol and lanosterol. Aside from triterpenoids, linoleic acid (C_18_), nonacosane (C_29_), hentriacontane (C_31_) and α-tocopherol were also detected (Table 2, Supplementary Fig. 3).

**Table 1:** Components of *Brosimum alicastrum* cutin identified using GC–MS

ID	RT	Name	Mean peak area ± S.E (× 10^6^)	Class	C:D	SI	RSI	Prob
1	30.41	Hexadecanoic acid*	260.0 ± 42.8	Fatty acid	16:0	889	904	65
2	30.54	11-Hexadecenoic acid*	11.3 ± 4.15	Fatty acid	16:1	855	907	49
3	31.55	Hexadecanoic acid**	24.3 ± 3.46	Fatty acid	16:0	845	892	96
4	32.25	15-Methyl hexadecanoic acid*	21.4 ± 2.99	Fatty acid	16:1	811	823	45
5	33.51	2-Hydroxy hexadecanoic acid***	7.1 ± 1.25	Fatty acid	16:0	773	843	85
6	34.14	9-Octadecenoic acid*	146.0 ± 34.5	Fatty acid	18:1	892	896	13
7	34.23	11-Octadecenoic acid*	12.1 ± 2.20	Fatty acid	18:1	837	859	10
8	34.49	8,11-Octadecadienoic acid*	217.0 ± 36.3	Fatty acid	18:2	904	921	23
9	35.00	9,12,15-Octadecatrienoic acid*	57.7 ± 9.81	Fatty acid	18:3	860	864	61
10	37.44	Eicosanoic acid*	17.6 ± 4.23	Fatty acid	20:0	838	878	84
11	40.58	Docosanoic acid*	31.0 ± 5.55	Fatty acid	22:0	789	829	88
12	41.96	Tricosanoic acid**	13.5 ± 3.95	Fatty acid	23:0	792	848	95
13	42.72	2-Hydroxy docosanoic acid***	14.7 ± 2.53	Fatty acid	22:0	750	805	87
14	43.49	Tetracosanoic acid*	5.4 ± 1.51	Fatty acid	24:0	720	786	81
15	44.09	2-Hydroxy tricosanoic acid***	9.6 ± 2.08	Fatty acid	23:0	683	777	71
16	44.68	2-Hydroxy hexacosanoic acid**	17.4 ± 4.44	Fatty acid	26:0	718	814	87
17	45.41	2-Hydroxy tetracosanoic acid***	25.4 ± 4.34	Fatty acid	24:0	759	828	94
18	46.68	2-Hydroxy pentacosanoic acid***	2.7 ± 1.02	Fatty acid	25:0	614	719	64
19	47.23	Octacosanol**	5.8 ± 2.15	Fatty alcohol		666	803	66
20	49.72	α-Tocopherol**	2.5 ± 1.12	Antioxidant		563	714	66
21	52.04	α-Sitosterol**	54.8 ± 16.3	Triterpenol		785	798	87
22	52.69	α-Amyrin**	11.6 ± 3.94	Triterpenol		689	839	51
23	53.64	Lupeol acetate	16.5 ± 5.01	Triterpenol		597	658	28

ID corresponds to the numbered peaks in Supplementary Fig. 2; RT is the retention time; and C:D is the carbon:double bond ratio of fatty acid components. Asterisks indicate whether the compound was detected as a *methyl ester derivative, **trimethylsilyl (TMS) derivative or ***TMS methyl ester derivative. The mean peak area is based on 5 replicates and is normalised to the peak area of the heptadecanoic acid internal standard. SI is a direct matching factor between the spectrum of an unknown peak and the library spectrum, RSI is a reverse search matching factor ignoring any unknown peaks that are not present in the library spectrum. Library hits with SI and RSI values above 900 are an excellent match, 800–900 a good match, and < 600 a poor match. Prob. indicates the probability of a correct identification based on the quality of the match and also the uniqueness of the spectrum for that compound compared to all other spectra in the database

**Table 2:** Components of *Brosimum alicastrum* cuticular wax identified using GC–MS

ID	RT	Name	Mean peak area ± S.E. (× 10^6^)	Class	SI	RSI	Prob
1	34.46	8,11-Octadecadienoic acid*	7.8 ± 1.10	Fatty acid	825	877	21
2	44.13	Nonacosane	18.2 ± 4.55	Alkane	808	861	23
3	46.78	Hentriacontane	22.5 ± 4.92	Alkane	790	852	16
4	50.02	α-Tocopherol	8.5 ± 1.44	Antioxidant	667	840	74
5	52.07	α-Sitosterol**	49.3 ± 13.2	Triterpenol	743	763	76
6	52.61	Lanosterol**	17.1 ± 5.06	Triterpenol	681	821	60
7	52.71	α-Amyrin	8.6 ± 2.34	Triterpenol	650	810	37
8	53.13	Cycloartenol acetate	11.1 ± 2.30	Triterpenol	654	744	16
9	53.33	Lanosterol**	32.8 ± 7.41	Triterpenol	705	839	62
10	53.65	9,19-Cyclolanostane-3,7-diol	24.1 ± 4.83	Triterpenol	612	695	13
11	54.06	γ-Sitosterol	108.0 ± 13.7	Triterpenol	856	875	61
12	55.4	9,19-Cyclolanost-7-en-3-ol	98.0 ± 13.8	Triterpenol	769	788	29
13	55.57	α-Amyrin	32.6 ± 9.11	Triterpenol	776	824	54
14	55.79	Cycloartenol	63.5 ± 11.1	Triterpenol	814	928	63
15	55.99	Lanosterol acetate	72.0 ± 9.93	Triterpenol	753	786	49
16	56.31	Lanosterol	208.0 ± 32.1	Triterpenol	782	786	29
17	56.48	24-Methylenecycloartanol	20.9 ± 4.81	Triterpenol	714	877	58
18	56.97	Lupeol	91.1 ± 16.2	Triterpenol	805	847	67
19	57.81	Unknown	16.4 ± 2.75	Triterpenol			

ID corresponds to the numbered peaks in Supplementary Fig. 3 and RT is the retention time. Asterisks indicate whether the compound was detected as a *methyl ester derivative or a **trimethylsilyl (TMS) derivative. The mean peak area is based on 5 replicates. Peaks were identified through spectral library matching. SI is a direct matching factor between the spectrum of an unknown peak and the library spectrum, RSI is a reverse search matching factor ignoring any unknown peaks that are not present in the library spectrum. Library hits with SI and RSI values above 900 are an excellent match, 800–900 a good match, and < 600 a poor match. Prob. indicates the probability of a correct identification based on the quality of the match and also the uniqueness of the spectrum for that compound compared to all other spectra in the database

### Biophysical properties of the cutin and cuticular waxes

**Fig. 8:**
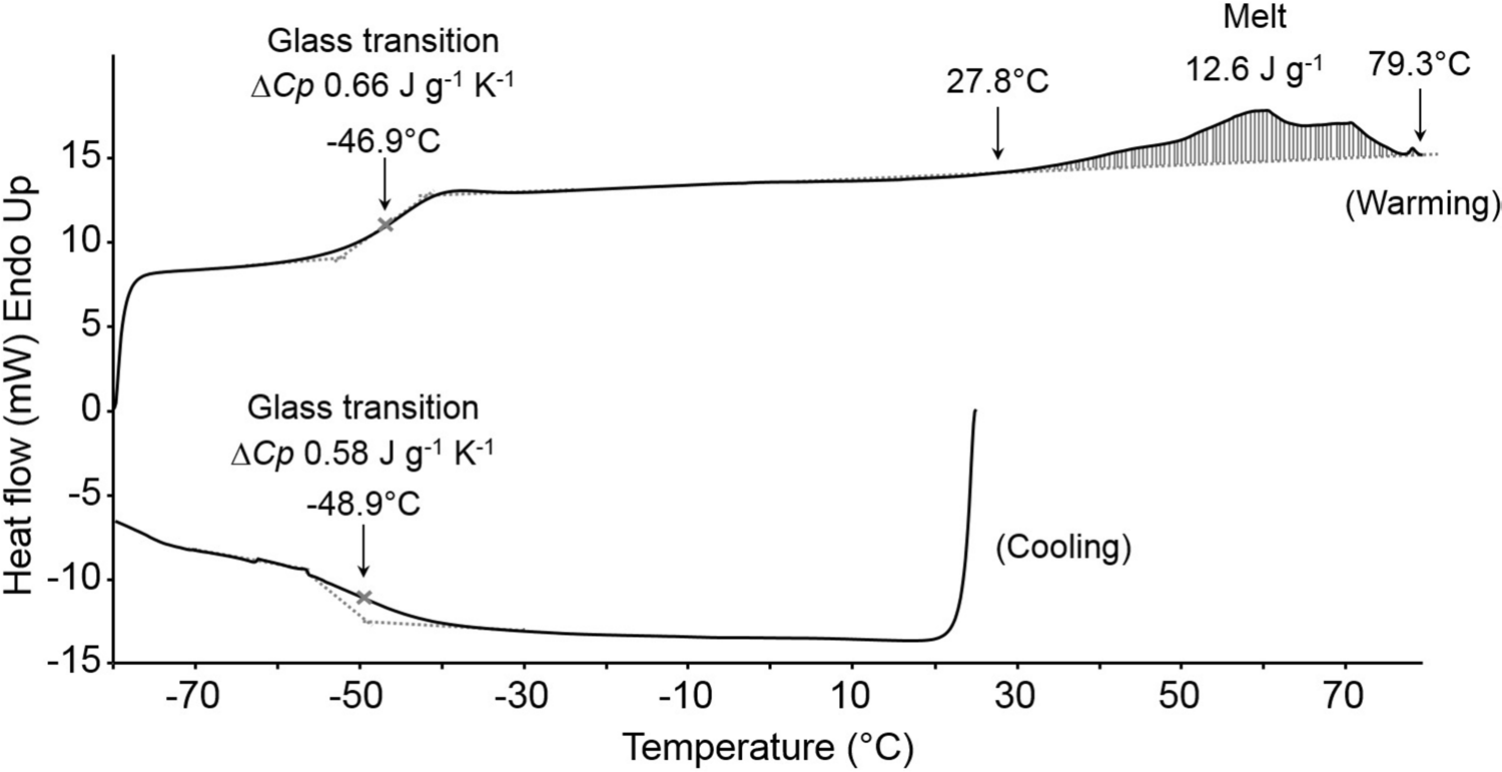
Representative differential scanning calorimetry thermograms of cuticular wax during cooling (25 to −80 °C) and warming (−80 to 80 °C) cycles. Arrows indicate the occurrence of glass transitions (*T*_g_) and melting events and their associated onset and end temperatures

No significant thermal event was noted for the cutin component, however, during the cooling cycle a glass transition for lipid was observed for the cuticular wax (Fig. 8). This transition temperature (*T*_g_) was −49.9 ± 1.4 °C with an average heat capacity (Δ*Cp*) of 0.50 ± 0.05 J g^−1^ °C^−1^. Similar results were obtained for the warming cycle where cutin showed no significant thermal event. Two significant thermal transitions, however, were noted for the cuticular wax. The first was a glass transition at -47.7 ± 0.9 °C and an average Δ*Cp* of 0.56 ± 0.09 J g^−1^ °C^−1^. The second a complex of endothermic transitions indicating the melting of waxes in the cuticular membrane. There are at least three prominent overlapping peaks indicating the melting of multiple lipid components. An integrated peak analysis showed the onset temperature for these melts was 26.4 ± 5.0 °C with an end temperature of 78.2 ± 1.2 °C. The average of total and fresh-weight-normalised enthalpies for this complex transition were 382.5 ± 149.4 mJ and 18.0 ± 10.0 J g^−1^, respectively.

## Discussion

### A recalcitrant seed with exceptional resistance to desiccation

Previous authors have reported *B. alicastrum* seeds as desiccation sensitive, but no physiological evidence for this has been presented (Pence 1995; Gillespie et al. 2004; Román et al. 2012). We demonstrate that *B. alicastrum* seeds combine the traits of large size (3.07 ± 0.44 g), high moisture content at shedding (> 50%) and relatively rapid germination that are typical of desiccation-sensitive species (Tweddle et al. 2003; Pritchard et al. 2004). Germination of *B. alicastrum* seeds was recorded during storage at temperatures above 15 °C, indicating that fresh seeds contain sufficient water and do not require an exogenous supply of water to germinate, which is another characteristic of desiccation-sensitive seeds (Berjak and Pammenter 2013). A probabilistic model to predict desiccation sensitivity based on the seed coat ratio returned a value of *P* = 0.97, which suggests that *B. alicastrum* seeds are likely to be desiccation sensitive. Previously, Daws et al. (2006) reported that the model wrongly classified *B. alicastrum* seeds as desiccation sensitive (*P* = 0.924), but our prediction of desiccation sensitivity is supported by a loss of germination over time in seeds equilibrated at either 5 or 15% RH. That this loss of germinability is not due to ageing alone is shown by the retention of viability in seeds held at 75% RH. We show that despite being desiccation sensitive, the seeds are able to survive for almost a year in a hydrated but quiescent state prior to germination with both seed water content and germination at values very similar to fresh seed when maintained at a temperature of 15 °C and 75% RH. This represents a formidable control of water loss for a large desiccation-sensitive seed.

### Anatomical, biochemical, and biophysical properties of the embryo cuticle

Anatomical characterisation of *B. alicastrum* seeds (Fig. 7) and the increased rate of water loss following experimental scarification (Figs. 4 and 5) suggest that the ability of the embryo to exert exceptional control of water loss is due to an impermeable cuticle covering the embryo’s epidermis. Water loss from scarified seeds followed a negative exponential pattern typical for seeds of desiccation-sensitive species (Hill et al. 2010). Removal of the cuticular wax accelerated water loss which indicates that structural feature(s) and not seed size or mass account for the control of water loss, in keeping with the results of a survey of tropical rainforest species in Australia (Hill et al. 2010).

Plant cuticles are a hydrophobic extracellular layer consisting of a lipid and hydrocarbon polymer known as cutin, impregnated with wax (Riederer and Schreiber 2001). Cuticular waxes are often responsible for the water barrier properties of the cuticle and their removal can result in a 100 to 1000-fold increase in permeability of isolated cuticles (Schreiber 2010). For intact *B. alicastrum* seed, we found that the rate of desiccation was ca. 12 times higher following the removal of cuticular wax, confirming that the cuticular waxes play a role in preventing water loss. Within the cuticle, there are two distinct layers of wax; the epicuticular wax which coats the outer surface of the cutin and the intracuticular wax that is embedded within the cutin polymer itself. The composition of these two wax layers varies but typically their major components are long-chain aliphatic compounds and cyclic compounds. It is the aliphatic components which determine the impermeability of the wax layer by forming tightly packed crystalline regions in the cuticle membrane that are impermeable (Bargel et al. 2006). The melt endotherms observed using DSC confirm that the cuticular waxes are in a crystalline state at room or physiological temperature, so may restrict water flow across the cuticle, which is consistent with other studies (Eckl and Gruler 1980; Aggarwal 2001). Studies of the permeance of leaf cuticles across several angiosperm families, including Moraceae, showed a marked increase in permeance at temperatures above 35 °C (Schreiber 2001; Riederer 2006). This temperature coincides with the onset of lipid melting observed in *B. alicastrum* cuticular wax, although Riederer (2006) proposed that changes in the volume of the polysaccharide component of cuticles may be responsible for increased permeance at higher temperatures rather than changes in the lipid fraction.

The cyclic structure of triterpenoids disrupts the crystalline structure of cuticular waxes making cuticles more permeable (Parsons et al. 2013). In general, such cyclic wax components accumulate in the intracuticular wax layer and are much less abundant in the epicuticular wax layer (Buschhaus and Jetter 2011). The soluble lipid fraction extracted from *B. alicastrum* comprises the epicuticular and intracuticular waxes and was dominated by triterpenoids, with only two very long-chain aliphatic compounds detected: nonacosane (C_29_) and hentriacontane (C_31_), although this may just reflect the relative abundance of epicuticular and intracuticular waxes and separate analysis of the wax layers is needed to assess how composition may relate to function.

Three types of cutins have been described in plants: (1) C_16_-type containing predominantly dihydroxyhexadecanoic acid, (2) C_18_-type containing mainly 9,10-epoxy-18-hydroxyoctadecanoic acid or 9,10,18-trihydroxyoctadecanoic acid, and (3) which is rich in C_18_ dicarboxylic acids (Yeats et al. 2012). *Brosimum alicastrum* cutin composition was distinct to these three types, comprising mainly saturated C_16_ and unsaturated C_18_ fatty acids, some very long-chain saturated fatty acids (C_20_–C_24_) and 2-hydroxy fatty acids (C_16_–C_26_). Long chain 2-hydroxy fatty acids are more often reported as monomers of suberin rather than cutin but are also major components of Arabidopsis cutin (Franke et al. 2005). Shao et al. (2007) reported that 2-hydroxy fatty acids were more abundant in the cutin of impermeable soybean seeds compared to that of permeable seeds and postulated that hydroxylated fatty acids reduce permeability by increasing the degree of cross-linking between cuticle components, thereby preventing the cuticle from cracking and water being lost through the cracks. Therefore, the cutin component of *B. alicastrum* cuticles may also contribute to the impermeability of the cuticle and control of water loss from the seed.

### Ecological implications of exceptional desiccation resistance

The formidable desiccation resistance of the embryo cuticle of *B. alicastrum* provides a mechanism whereby the seed can remain metabolically active, able to germinate without having to imbibe water first, whilst at the same time able to reduce water loss to such an extent that it can survive several months without dehydrating. This may explain the presence of the species across a broad range of rainfall gradients (Esquivel-Muelbert et al. 2017) and in both evergreen wet and seasonal dry forest (Pennington and Sarukhán, 2005). Following analyses of 1818 species distributions in Central America and the Western Amazon, Esquivel-Muelbert et al. (2017) suggest that most tree species’ distributions in the tropics are limited by water stress, and that communities in dry environments comprise a drought tolerant subset of species that could occur in wet environments. Accordingly, we take the broad distribution of *B. alicastrum* as evidence of an ability to overcome the challenges of drought rather than the ability to overcome the challenges associated with wet areas such as pathogens and pests (Spear et al. 2015). *Brosimum alicastrum* ranked in the top 12% of species with respect to cumulative water deficit across its range (Esquivel‐Muelbert et al. 2017). Two other *Brosimum* species of similar seed size but unknown desiccation traits, *B. utile* and *B. guianense*, ranked first and second. That is, they occupied the broadest range of cumulative water deficits across their range. Both have significantly larger ranges than *B. alicastrum*, encompassing Central America and South America, south of the Amazon (POWO 2022). We suggest that the pre-germination trait of desiccation avoidance plays an important role in the ability of *B. alicastrum* to colonise sites of elevated cumulative water deficit. We hypothesise that coupled with the increased post-germination survival advantages conferred by a large seed size (Baraloto et al. 2005; Poorter et al. 2008), desiccation avoidance enables the seed to defer germination until water deficits are reduced (Jurado and Flores 2005) and the conditions for post-germination survival improved.

The trait of desiccation tolerance is believed to be uncommon in large seeds because the time it would take a large, desiccated seed to imbibe to a water potential sufficient for germination (Kikuzawa and Koyama 1999) would render it unresponsive in a seasonal habitat where it would be most needed (Tweddle et al. 2003). Traits supporting resistance to desiccation could therefore enable large-seeded species to exploit the advantages of rapid shoot and root extension (Leishman et al. 2000) in areas of elevated cumulative water deficit, with the large cotyledons serving as a water pool for growth. The ten species with the most negative mean cumulative water deficits and lowest annual rainfall for their ranges in a survey by Esquivel-Muelbert et al. (2017) had seeds with a mass > 1 g, which suggests that large-seeded species may represent significant components of dry forest vegetation in Central America and North Western South America. Surprisingly, two of the ten species with the highest cumulative water deficits could be expected to be desiccation sensitive based on their morphology (*Sideroxylon obtusifolium* (Humb. ex Roem. and Schult.) T.D. Penn., *Pradosia colombiana* (Standl.) Penn. ex T.J. Ayers and Boufford), and three of the species occupying habitats with the lowest annual rainfall could be expected to be desiccation sensitive (*S. obtusifolium*, *P. colombiana*, *Ximenia americana* L.). It may therefore be that there are other large-seeded desiccation-sensitive species occupying seasonal dry habitats in the tropics whose desiccation resistance has simply not been documented and that the assumption that habitats with high cumulative water deficits are dominated by species that are desiccation tolerant or small seeded is an artefact of limited sampling.

Whilst recent analyses have reinforced that the trait of seed desiccation sensitivity is mainly found in tropical tree species (Wyse and Dickie 2017; Subbiah et al. 2019b), they also highlight that species with desiccation-sensitive seeds occur across diverse temperate habitats. For example, the genus *Quercus* (Fagaceae) contains a number of temperate tree species with desiccation-sensitive seeds (Subbiah et al. 2019b). Variation in drying rate between the seeds of nine *Quercus* species was found to be correlated with the density of vascular bundles in the scar and pericarp permeability, rather the cuticle, which in contrast to our findings with *B. alicastrum* seeds, did not influence water flow (Xia et al. 2012). Seed dispersal in these *Quercus* species usually coincides with the end of the wet season or beginning of the dry season, so a low drying rate enables seed survival over extended (ca. 6 months) dry periods (Xia et al. 2012). Seeds of *Swartzia langsdorffii* (Fabaceae), which are desiccation sensitive, have also been shown to remain viable in the soil for up to 7 months during the dry season in SE Brazil without significant decrease in seed water content (Vaz et al. 2016). The formidable and apparently exceptional control over water loss of *B. alicastrum* may, with further study, represent a trait that is widely distributed across species occupying areas of high cumulative water deficit. Understanding and documenting this trait related to pre-germination survival could therefore contribute greatly to our understanding not only of how species assemble but also how they will respond to changing precipitation regimes associated with climate change. For example, our study of *B. alicastrum* suggests that changes in precipitation regime associated with climate change across its ranges might have little impact on pre-germination survival of this species’ seeds. Therefore, pre-germination survival is unlikely to be a major factor in limiting the future distribution of *B. alicastrum*.

## Supplementary Information

Below is the link to the electronic supplementary material.

Supplementary file1 (PDF 271 KB)

## Data Availability

The data that support the findings of this study are available from the corresponding author upon reasonable request.
