# Supplementary material for: Exceptional desiccation resistance in recalcitrant seeds of Brosimum alicastrum may confer ecological advantage
Source: Planta. 2026 Jan 3;263(2):36. doi: 10.1007/s00425-025-04910-y (PMC12764665; doi:10.1007/s00425-025-04910-y)

Supplementary Table 1. Fresh weight (FW), dry weight (DW) and moisture content (MC; % FW basis) of the embryo and seed coat of 10 individual *Brosimum alicastrum* seeds. The seed coat ratio (SCR) was calculated as seed coat DW / whole seed DW. The P-value is the probability that a seed is recalcitrant based on the methodology of Daws et al., 2006. P > 0.5 indicates that a seed is more likely to be recalcitrant.

| | Embryo | | | Seed coat | | | Whole seed | | | | |
|---|---|---|---|---|---|---|---|---|---|---|---|
| **Seed** | **FW (g)** | **DW (g)** | **% MC** | **FW (g)** | **DW (g)** | **% MC** | **FW (g)** | **DW (g)** | **% MC** | **SCR** | **P value** |
| 1 | 3.1534 | 1.3164 | 58.2545 | 0.0679 | 0.0555 | 18.2273 | 3.2213 | 1.3719 | 57.4105 | 0.0405 | 0.9677 |
| 2 | 2.5780 | 1.1525 | 55.2959 | 0.0385 | 0.0331 | 13.9886 | 2.6165 | 1.1856 | 54.6887 | 0.0279 | 0.9672 |
| 3 | 2.4384 | 1.0031 | 58.8633 | 0.0342 | 0.0291 | 14.6999 | 2.4725 | 1.0322 | 58.2533 | 0.0282 | 0.9616 |
| 4 | 3.2201 | 1.3135 | 59.2089 | 0.0466 | 0.0391 | 15.9149 | 3.2667 | 1.3527 | 58.5918 | 0.0289 | 0.9715 |
| 5 | 2.1327 | 0.9255 | 56.6037 | 0.0358 | 0.0304 | 15.1591 | 2.1686 | 0.9559 | 55.9191 | 0.0318 | 0.9565 |
| 6 | 3.8833 | 1.7609 | 54.6538 | 0.0537 | 0.0461 | 14.2538 | 3.9370 | 1.8070 | 54.1023 | 0.0255 | 0.9811 |
| 7 | 2.4021 | 0.9750 | 59.4115 | 0.0481 | 0.0427 | 11.3098 | 2.4502 | 1.0176 | 58.4672 | 0.0419 | 0.9543 |
| 8 | 3.0018 | 1.3776 | 54.1077 | 0.0379 | 0.0333 | 12.0844 | 3.0397 | 1.4109 | 53.5837 | 0.0236 | 0.9747 |
| 9 | 2.7919 | 1.0763 | 61.4480 | 0.0375 | 0.0328 | 12.5033 | 2.8294 | 1.1092 | 60.7992 | 0.0296 | 0.9639 |
| 10 | 2.6882 | 1.0008 | 62.7704 | 0.0360 | 0.0321 | 10.7838 | 2.7242 | 1.0329 | 62.0838 | 0.0311 | 0.9603 |
| Mean | 2.8290 | 1.1902 | 58.0618 | 0.0436 | 0.0374 | 13.8925 | 2.8726 | 1.2276 | 57.3900 | 0.0309 | 0.9659 |
| S.D. | 0.5059 | 0.2568 | 2.8767 | 0.0107 | 0.0084 | 2.2806 | 0.5122 | 0.2615 | 2.8237 | 0.0059 | 0.0083 |
| S.E. | 0.1600 | 0.0812 | 0.9097 | 0.0034 | 0.0027 | 0.7212 | 0.1620 | 0.0827 | 0.8929 | 0.0019 | 0.0026 |

Supplementary Fig. 1. Cumulative germination of *Brosimum alicastrum* seeds at constant temperatures (5 – 40°C). Data points represent the means of three replicates of 15 seeds. Logistic regression curves were fitted to the data.

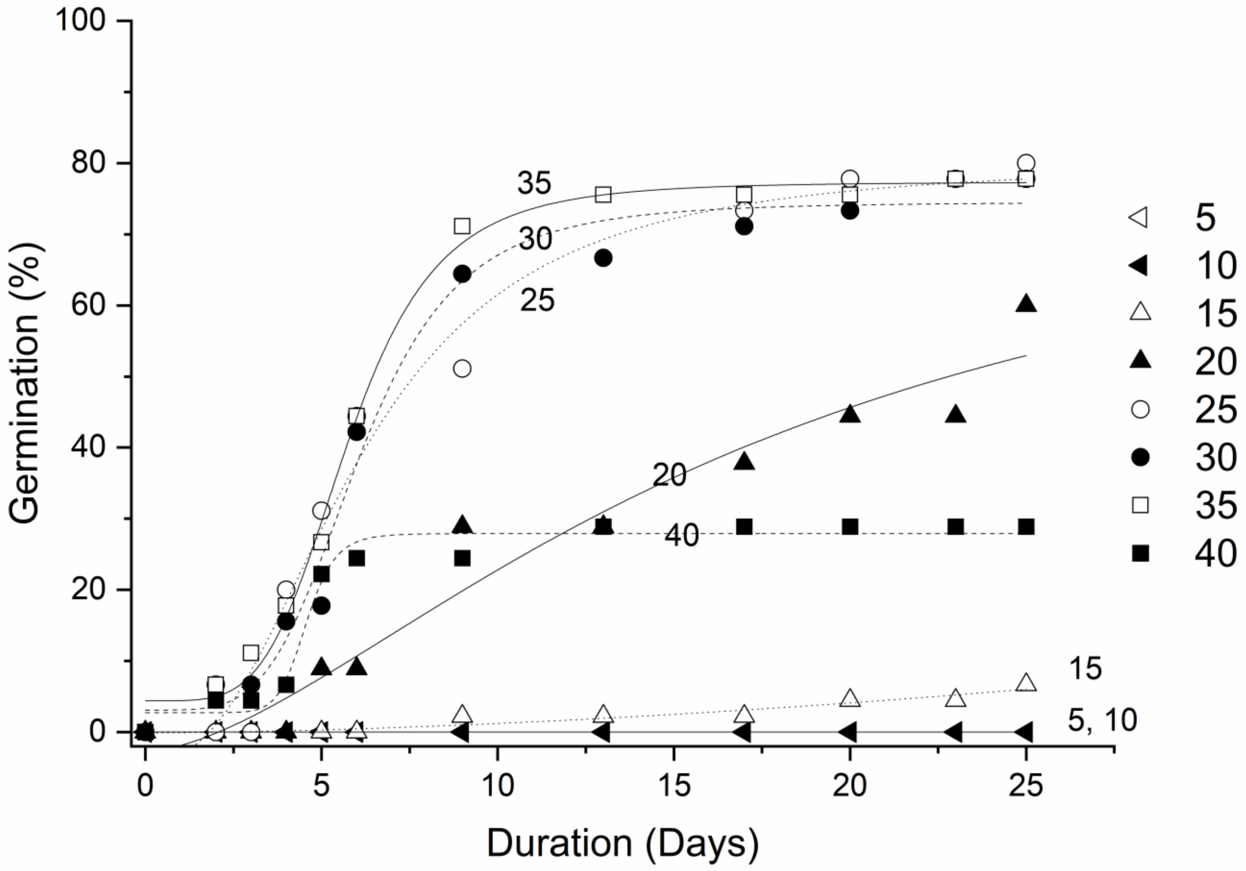

Supplementary Fig. 2. GC-MS chromatogram of cutin components. Numbers correspond to compounds listed in Table 1. IS indicates the internal standard (heptadecanoic acid) peak.

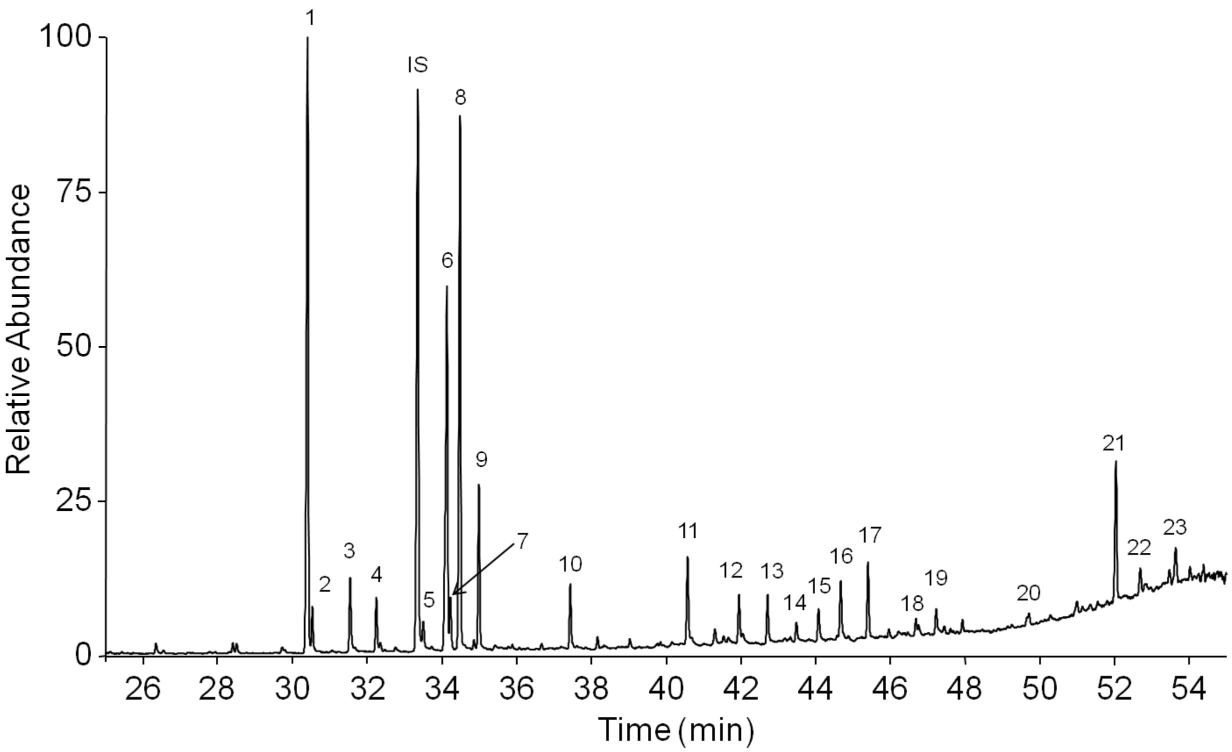

Supplementary Fig. 3. Chromatogram of cuticular wax components. Numbers correspond to compounds listed in Table 2.

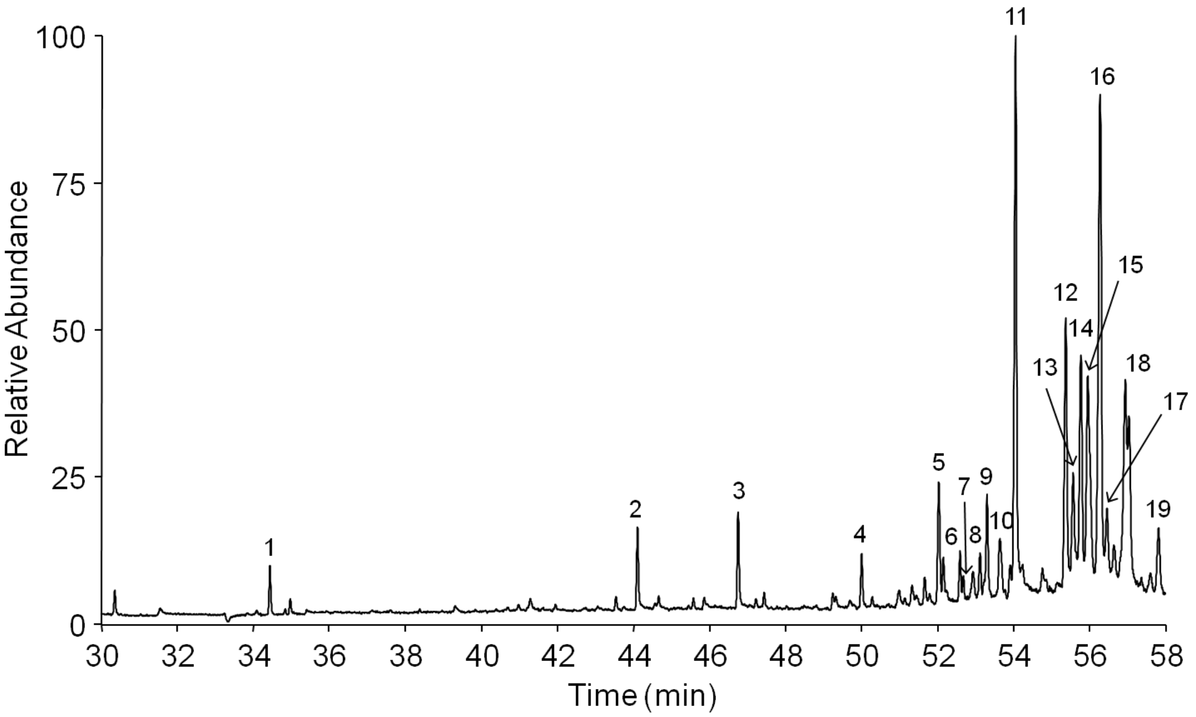

Supplement: Supplementary file 1 — Supplementary file1 (PDF 271 KB) [file 425_2025_4910_MOESM1_ESM.pdf]
